# Are Scores on English and French Versions of the PHQ-9 Comparable? An Assessment of Differential Item Functioning

**DOI:** 10.1371/journal.pone.0052028

**Published:** 2012-12-14

**Authors:** Erin Arthurs, Russell J. Steele, Marie Hudson, Murray Baron, Brett D. Thombs, (CSRG) Canadian Scleroderma Research Group

**Affiliations:** 1 Department of Psychiatry, McGill University, Montréal, Québec, Canada; 2 Department of Mathematics and Statistics, McGill University, Montréal, Québec, Canada; 3 Department of Epidemiology, Biostatistics, and Occupational Health, McGill University, Montréal, Québec, Canada; 4 Department of Medicine (Division of Rheumatology), McGill University, Montréal, Québec, Canada; 5 Department of Educational and Counselling Psychology, McGill University, Montréal, Québec, Canada; 6 School of Nursing, McGill University, Montréal, Québec, Canada; 7 Lady Davis Institute for Medical Research, Jewish General Hospital, Montréal, Québec, Canada; Federal University of Rio de Janeiro, Brazil

## Abstract

**Background:**

Medical research increasingly utilizes patient-reported outcome measures administered and scored in different languages. In order to pool or compare outcomes from different language versions, instruments should be measurement equivalent across linguistic groups. The objective of this study was to examine the cross-language measurement equivalence of the Patient Health Questionnaire-9 (PHQ-9) between English- and French-speaking Canadian patients with systemic sclerosis (SSc).

**Methods:**

The sample consisted of 739 English- and 221 French-speaking SSc patients. Multiple-Indicator Multiple-Cause (MIMIC) modeling was used to identify items displaying possible differential item functioning (DIF).

**Results:**

A one-factor model for the PHQ-9 fit the data well in both English- and French-speaking samples. Statistically significant DIF was found for 3 of 9 items on the PHQ-9. However, the overall estimate in depression latent scores between English- and French-speaking respondents was not influenced substantively by DIF.

**Conclusions:**

Although there were several PHQ-9 items with evidence of minor DIF, there was no evidence that these differences influenced overall scores meaningfully. The PHQ-9 can reasonably be used without adjustment in Canadian English- and French-speaking samples. Analyses assessing measurement equivalence should be routinely conducted prior to pooling data from English and French versions of patient-reported outcome measures.

## Introduction

Health-related patient-reported outcomes (HR-PROs) assess patient health based on patient perspectives. HR-PROs may reflect complex constructs, such as health-related quality of life (HRQL), or narrower constructs, such as pain, fatigue or depressive symptoms [1]–[3]. Assessment of HR-PROs has been emphasized in recent years, especially among patients with chronic diseases [4], and this has been reflected in initiatives aimed at improving measurement quality and operationalization in research and clinical practice, such as the PROMIS initiative [5] and OMERACT in rheumatology [6]. Recently, the COSMIN checklist (Consensus based Standards for the selection of health status Measurement INstruments) was developed to establish general criteria for assessing the methodological quality of studies that evaluate measurement properties of HR-PROs [7].

In recent years, international collaboration, and, thus, interest in HR-PRO research in the context of multinational studies, has grown [8]. HR-PROs are increasingly translated and used in diverse linguistic and cultural settings [9]. As described in the COSMIN checklist [7], the cross-linguistic or cross-cultural equivalence of HR-PRO measures is an important consideration for instruments that will be administered in more than one language or for comparisons of results across linguistic or cultural settings. When HR-PRO measures are measurement equivalent across groups, individuals with similar levels of the construct being measured (e.g., symptoms of depression) will score similarly on each item of the measure and scoring metrics can be considered equivalent across the groups [10], [11]. Alternatively, differential item functioning (DIF) is said to occur when members of one group are more or less likely to endorse a particular item or symptom than members of the other group with the same level of the latent trait that is being measured [12]. DIF is assessed by evaluating whether there are differences in scores of individual items across groups after controlling for levels of the construct being measured. When DIF occurs for an item, it is assumed that item scores are influenced by a group characteristic other than the construct being measured. When measures are translated and administered in different linguistic or cultural groups, DIF may occur because of imperfect translation or because of cultural factors that may influence interpretation of item meaning. Valid comparisons of scores across groups or combining scores from different cultural or linguistic groups in a single analysis require equivalent measurement metrics and the lack of substantive DIF [13].

Depression is an important determinant of HRQL among patients with chronic illnesses [14]. The Patient Health Questionnaire-9 (PHQ-9) [15] is an easily administered and scored self-report measure of the frequency and distress from depressive symptoms with 9 symptom items that map directly onto the Diagnostic and Statistical Manual for Mental Disorders, Fourth Edition criteria for a major depressive episode [16]. The PHQ-9 has been shown to be accurate for identifying cases of major depression in a range of settings and patient populations [17]–[19]. It is available in 49 languages [20], including English and French. No studies, however, have tested whether scores on the PHQ-9 are comparable across different language versions. In Canada, scores from English and French versions of HR-PRO measures are routinely combined in analyses, but there are few examples where measurement equivalence or DIF have been assessed for any HR-PRO.

The objective of this study was to conduct a DIF analysis to determine whether PHQ-9 scores are equivalent between English and French versions. To do this, we compared item responses on the PHQ-9 from English- and French-speaking scleroderma patients enrolled in the Canadian Scleroderma Research Group Registry, a pan-Canadian collaboration that includes both English- and French-speaking patients. Scleroderma, or systemic sclerosis (SSc), is an autoimmune disease characterized clinically by thickening and fibrosis of the skin and by the involvement of internal organs, most commonly the lungs, gastrointestinal tract and heart [21]. High levels of depressive symptoms are common in patients with SSc and are related to overall SSc disease severity, as well as specific medical symptoms [22].

## Methods

### Ethics Statement

This study was approved by the research ethics committee of Mcgill University. All patients provided informed written consent, and the research ethics board of each participating center approved the data collection protocol.

### Patient Sample and Procedures

The study sample consisted of patients enrolled in the Canadian Scleroderma Research Group (CSRG) Registry from its initiation in September 2004 through January 2011. The CSRG includes fifteen centers from across Canada. Patients in the CSRG Registry must be ≥18 years old at the time of enrolment, diagnosed with SSc by a CSRG rheumatologist, and fluent in French or English. At annual Registry visits, patients undergo extensive clinical evaluations and complete a series of self-report questionnaires. Registry patients were included in the present analysis if they were missing 2 or fewer PHQ-9 items. Patients were not included if they were missing disease related variables, including physician rated disease severity, modified Rodnan skin score, number of gastrointestinal symptoms, breathing problems and diffuse status.

### Measures

CSRG Registry data used in the present study included self-reported sociodemographic variables, physician- and patient-reported disease-related variables, and symptoms of depression, as measured by the PHQ-9.

#### Symptoms of depression

The PHQ-9 is a 9-item self-report measure in which respondents rate the frequency of depression symptoms over the past 2 weeks on 0–3 Likert-type scales (*not at all* to *nearly every day)*, with higher scores on each item reflecting a greater amount of time in which the patient was bothered by the symptom. The total score ranges from 0–27 with the standard cutoff threshold for “moderate” depression severity as a score of ≥10 [23]. It has recently been found to have good validity and reliability amongst an SSc patient group [24], although that study used PHQ-9 data in English and French and did not assess potential differences in English and French versions. English and Canadian French versions of the PHQ-9 were retrieved from the Pfizer Patient Health Questionnaire Screeners website [20]. No information is available on the site on how the PHQ-9 was translated and adapted into non-English versions.

#### Disease-related variables

SSc disease characteristics were obtained via patients’ medical histories and examinations by study physicians. SSc global disease severity was rated by study physicians on a 0–10 numerical rating scale (*no disease* to *very severe disease*), a scale that has been shown to be a valid measure of severity in SSc [25]. Limited skin disease was defined as skin involvement distal to the elbows and knees with or without face involvement and diffuse skin disease as skin involvement proximal to the elbows and knees and/or involving the trunk [26]. Extent of skin involvement was assessed using the modified Rodnan skin score ranging from 0 to 51 [27]. Breathing problems were rated by patients on a 0–10 numerical rating scale (*no shortness of breath* to *very severe shortness of breath*) [28]. The number of gastrointestinal symptoms was determined by patient report from a checklist that included weight loss, anorexia, dysphagia, reflux, pyrexia, choking at night, early satiety, bloating, nausea/vomiting, constipation, diarrhea, malabsorption, fecal incontinence, antibiotics for bacterial overgrowth, and hyperalimentation [22].

### Statistical Analyses

We compared all demographic and clinical characteristics between language groups using independent sample t-tests for continuous variables and chi-square tests for categorical variables.

Ideally for DIF assessment, the simplest structure with reasonable fit will be used. The PHQ-9 has been shown to be a single-dimensional measure of depressive symptoms in a primary care setting [29]. A two-factor model representing cognitive/affective and somatic factors has also been proposed in, for example, spinal cord injury [30]–[31] and cardiovascular disease [32]. Thus, we tested a single-factor model, as well as a two-factor model that included items 1, 2, 6, 7 and 9 (*lack of interest, depressed mood, worthlessness, concentration problems, suicide ideation*) on a cognitive/affective factor and items 3–5 and 8 (*sleep difficulties, fatigue, appetite problems, psychomotor agitation or retardation*) on a somatic factor.

Confirmatory factor analysis (CFA) was conducted with Mplus version 7 [30] to assess the validity of the previously reported single-dimensional structure of the PHQ-9 in SSc. Item responses for the PHQ-9 are ordinal Likert data and were modeled as such. To do this, Mplus initially estimates item thresholds for ordinal outcome variables using maximum likelihood methods. These estimates are then used to estimate a polychoric correlation matrix. Model parameters are subsequently estimated with weighted least squares using the weighted least squares estimator with a diagonal weight matrix, robust standard errors, and a mean- and variance-adjusted chi-square statistic was used with delta parameterization [33]. Standard Mplus procedures for estimating models that included patients with missing data were used (full information maximum likelihood). A chi-square goodness-of-fit test and 3 fit indices were used to assess model fit, including the Tucker-Lewis Index (TLI, [34]) the Comparative Fit Index (CFI, [35]) and the Root Mean Square Error of Approximation (RMSEA, [36]). Since the chi-square test is highly sensitive to sample size and can lead to the rejection of well-fitting models, practical fit indices were emphasized [11]. Guidelines proposed by Hu & Bentler [37] suggest that models with TLI and CFI ≤.95 and the RMSEA ≤.06 are representative of good fitting models. Initially, CFA models were fit with data from English- and French-speaking patients separately, then a single model was fit with data from all patients combined. Chi-square difference tests are sometimes used to determine whether a less parsimonious model substantively improves fit compared to a simpler model. However, these procedures are highly sensitive to sample size. Thus, we used a procedure recommended by Cheung et al. [38] that involves comparing the change in goodness-of-fit indices, which are not affected by sample size, between two models to determine whether there are substantive differences in model fit. Consistent with Cheng’s recommendations, we compared the CFI between the single-factor and two-factor models with a difference of ≤0.01 indicative of substantively similar models [38].

**Figure 1:**
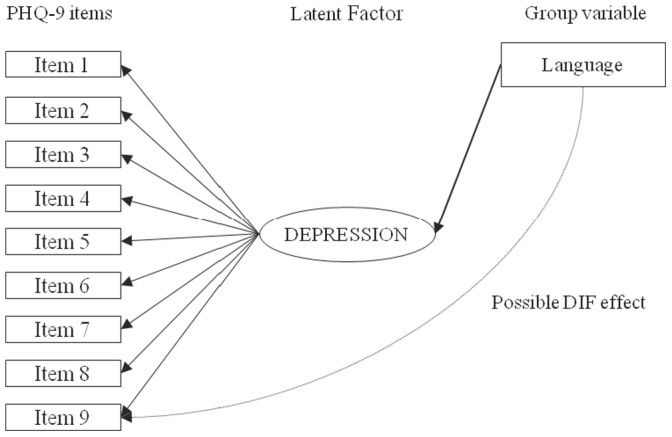
MIMIC model for Patient Health Questionnaire - 9 (PHQ-9). The base Multiple-Indicator Multiple-Cause (MIMIC) model consists of a confirmatory factor model with the additional direct effect of group (language) on the latent depression factor serving to control for group differences on the level of the latent depression factor. For differential item functioning (DIF) analysis, the direct effect of group on PHQ-9 items is assessed for each item separately by regressing items, one at a time, on language, controlling for any differences in the overall level of the latent depression factor between groups.

The Multiple-Indicator Multiple-Cause (MIMIC) model was utilized to determine if items of the PHQ-9 exhibited DIF for English- versus French-speaking patients. MIMIC models for DIF assessment are based on structural equation models, in which the group variable (English versus French) is added to the basic CFA model as an observed variable. Thus, the base MIMIC model consists of the CFA factor model with the additional direct effect of group on the latent factor, which serves to control for group differences on the level of the latent depression factor. Then, to assess potential DIF, in addition to regressing the latent factor on linguistic group, the direct effect of group on PHQ-9 items is assessed for each item separately, by regressing items, one at a time, on group (see Figure 1). Items are tested separately to determine if there is statistically significant DIF, represented by a statistically significant link in the model from group to the item, after controlling for any differences in the overall level of the latent depression factor between groups. If there is DIF for one or more items, the item with the largest magnitude of DIF is considered to have DIF, and the link between the linguistic group variable and that item is included in the model. Then, this procedure is repeated until none of the remaining items show significant DIF. Once all items with significant DIF are identified, the potential magnitude of DIF items collectively, identified via assessment of statistical significance, can be evaluated by comparing the difference on the latent factor between groups in the baseline CFA model and after controlling for DIF. Since the PHQ-9 consists of 9 items, Hochberg’s Sequential Method [39] was used to maintain a family-wise Type I error rate of α <0.05 for multiple item comparisons. CFA and DIF analyses were conducted using Mplus [33], and all other analyses were conducted using IBM SPSS Statistics 20 (Chicago, IL).

## Results

### Sample Characteristics

Table 1 shows the patient demographics and clinical characteristics of the sample. Of the sample of 960 patients, 77% (n = 739) were English-speaking patients and 87% (n = 839) were female. The mean (±standard deviation) age of the sample was 56.6±11.5 years; 51% (n = 494) of patients completed some post-secondary education, and 70% (n = 672) were married or living as married. Approximately 31% (n = 301) of patients had diffuse SSc; the mean physician-rated global severity score was 2.8±2.2 and mean total skin score was 9.7±9.0. The mean number of gastrointestinal problems was 3.6±3.0, and the mean breathing problems score on a 0–10 scale was 2.0±2.6. The only statistically significant difference between English- and French-speaking patients was physician-rated global disease severity (p = .01), although the magnitude of the difference was small (standardized mean difference = 0.23).

**Table 1:** Clinical Characteristics.

	Overall (N = 960)	English-speaking Patients (N = 739)	French-speaking Patients (N = 221)	p Value
**Sociodemographic Characteristics**				
Age in years, *mean (standard deviation)*	56.6 (11.5)	56.3 (11.8)	57.5 (10.2)	0.17
Female sex, *n (%)*	839 (87.4)	643 (87.0)	196 (88.7)	0.57
Education, *n (%)*:				
< High school	461 (48.3)^a^	364 (49.6)^b^	97 (43.9)	0.15
≥ High school	494 (51.7)^a^	370 (50.4)^b^	124 (56.1)	
Not reported	5 (0.5)^a^	5 (0.7)^b^	0 (0.0)	
Marital status, *n (%)*	672 (70.1)^c^	525 (71.1)^d^	147 (66.5)	0.21
**Clinical Characteristics**				
Breathing problems (1–10), *mean (SD)*	2.0 (2.6)	1.9 (2.5)	2.2 (2.8)	0.20
Physician-rated global disease severity (1–10), *mean (SD)*	2.8 (2.2)	2.7 (2.1)	3.2 (2.4)	0.01
Modified Rodnan total skin score, *mean (SD)*	9.7 (9.0)	9.7 (8.9)	10.0 (9.1)	0.64
Number of gastrointestinal symptoms, *mean (SD)*	3.6 (3.0)	3.6 (3.0)	3.8 (3.0)	0.25
Diffuse SSc, *n (%)*	301 (31.4)	225 (30.4)	76 (34.4)	0.28
**PHQ-9 Characteristics**				
Score of 10 or higher, *n (%)*	202 (21.4)^e^	153 (21.1)^f^	49 (22.4)^g^	0.64
Total Score, *mean (SD)*	6.0 (5.3)^e^	5.9 (5.3)^f^	6.3 (5.5)^g^	0.28

a percentage based on n = 955 patients overall.

b percentage based on n = 734 English-speaking patients.

c percentage based on n = 959 patients overall.

d percentage based on n = 738 English-speaking patients.

e mean based on n = 945 patients overall.

f mean based on n = 726 English-speaking patients.

g mean based on n = 219 French-speaking patients.

The mean total PHQ-9 score for the 945 patients with complete PHQ-9 data was 6.0±5.3. Mean scores for English and French versions were 5.9±5.3 and 6.3±5.5, respectively (p = 0.28). Just over 20% of the total sample scored ≥10 on the PHQ-9 (n = 202, 21.0%), including 21.1% of English-speaking patients (n = 153) and 22.4% of French-speaking patients (n = 49; p = 0.64). Of the 15 patients with missing PHQ-9 data, 10 patients were missing 1 item, and 5 patients were missing 2 items.

### Confirmatory Factor Analysis

Single- and two-factor structures were first assessed for each sample separately. For the single-factor, there was excellent fit in each sample (English: χ^2^ (18) = 119.4, p<0.001, CFI = 0.97, TLI = 0.99, RMSEA = 0.09, Cronbach’s α = 0.87; French: χ^2^ (17) = 32.6, *P* = 0.013 CFI = 0.99, TLI = 0.99, RMSEA = 0.07, Cronbach’s α = 0.86). Fit was similar for the two groups for the two-factor structure (English: χ^2^ (18) = 135.2, *P*<0.001, CFI = 0.97, TLI = 0.98, RMSEA = 0.09; French: χ^2^ (18) = 29.2, p = 0.046 CFI = 0.99, TLI = 0.99, RMSEA = 0.05).

When data for the English- and French speaking patients were combined together in one model, the single-factor model continued to fit well based on standard fit indices (χ^2^ (26) = 208.1, p*<*0.001, CFI = 0.96; TLI = 0.98, RMSEA = 0.09; Cronbach’s α = 0.87), as did the two-factor model (χ^2^ (25) = 177.1, p*<*0.001, CFI = 0.97; TLI = 0.98, RMSEA = 0.08). There was no substantive difference between the single-factor and two-factor models in terms of standard fit indices (CFI, TLI, RMSEA) to suggest that the two-factor model provided better fit. Furthermore, the high correlation between the cognitive/affective and somatic factors (0.91) suggested a high level of redundancy. Thus, all subsequent analyses were done with the single-factor model. Table 2 shows the baseline CFA single-factor model parameters and fit characteristics, with data from both English- and French-speaking patients combined, prior to assessing DIF. Factor loadings ranged from 0.64 to 0.87.

**Table 2:** Baseline and Final Model Characteristics with Summary Measures Of Model Fit.

Model Items	Model Estimate with no DIF (95% Confidence Interval)	Model Estimate with DIF (95% Confidence Interval)
1. Lack of interest	0.87 (0.84–0.90)	0.87 (0.84–0.90)
2. Depressed mood	0.87 (0.84–0.92)	0.87 (0.84–0.92)
3. Sleeping problems	0.65 (0.60–0.70)	0.65 (0.60–0.70)
4. Fatigability	0.80 (0.76–0.83)	0.80 (0.76–0.83)
5. Appetitive problems	0.64 (0.59–0.69)	0.64 (0.59–0.69)
6. Negative feelings about self	0.87 (0.84–0.89)	0.87 (0.84–0.89)
7. Concentration problems	0.77 (0.73–0.81)	0.77 (0.73–0.81)
8. Psychomotor agitation/retardation	0.73 (0.68–0.78)	0.73 (0.68–0.78)
9. Suicidal ideation	0.77 (0.71–0.83)	0.77 (0.71–0.83)
**Item displaying DIF regressed on language:**		
2. Depressed mood		0.25 (0.12–0.39)
3. Sleeping problems		0.28 (0.13–0.42)
6. Negative feelings about self		0.21 (0.07–0.35)
**Regression of Language on Depression Latent Factor:**		
Reference group: French Speaking Patient	−0.056 (−0.215–0.103)	−0.169 (−0.334– −0.004)
**Latent Variable Residuals:**		
Depression latent factor	1.00–	1.00–
**Latent Variable Intercepts:**		
Depression latent factor	0.00–	0.00–
**Model Fit Summary:**		
Model Chi-Square (df), P-value	208.1 (26), p*<*0.001	188.0 (24), p*<*0.001
CFI	0.963	0.967
RMSEA	0.085	0.084
TLI	0.981	0.982

#### Differential item functioning

As shown in Table 2, prior to accounting for DIF, the level of the latent depression factor was −0.056 standard deviations (lower) for English-speaking compared to French-speaking patients, although this was not statistically significant (95% confidence interval for English-speakers, −0.215 to 0.103). Statistically significant DIF was identified in three items (item 2-*depressed mood*; p<0.01, item 3-*sleeping problems*; p<0.01 and item 6-*negative feelings about self*; p<0.01). For each of these items, English-speaking patients endorsed the item at a somewhat higher level that would have been expected based on their latent depression factor level. However, the magnitude of DIF was small and did not substantively or significantly influence differences between English- and French-speaking patients on the latent depression variable. After adjusting for DIF, the difference between English- and French-speaking patients on the latent depression variable was −0.169 (95% confidence interval, −0.334 to −0.004). In order to test whether any differences in clinical characteristics between the two groups may have influenced the DIF analysis, we refit the MIMIC model, controlling for demographic and clinical characteristics (global disease severity, diffuse skin disease, Rodnan skin score, breathing problems, number of gastrointestinal symptoms). There was no improvement in fit statistics for the model with the added covariates, and their inclusion did not alter the results of the DIF analysis.

## Discussion

Self-report questionnaires are increasingly used in health research to assess patient-reported outcomes and are commonly administered in more than one language. In order to compare results from these measures between respondents from different language groups or to pool data from different language versions of a measure, the cross-linguistic equivalence of scores should be established. The main finding of this study was that three items on the PHQ-9 exhibited statistically significant DIF in a sample of SSc patients who completed the measure in English or French. However, the magnitude of DIF for each item was small, and the effect on depression scores was negligible. These results suggest that English- and French-language versions of the PHQ-9 can be used among patients with SSc without concern that outcomes will be substantively influenced by differences in scoring metrics between the two versions.

Although the PHQ-9 is available in 49 languages, it has not been validated in all of these languages. When studies have validated the PHQ-9 in different languages [40]–[43], results have confirmed that the PHQ-9 continues to be a valid measure of depression after translation. This does not, however, establish that depression symptom scores in one language are equivalent to scores in another language. In Canada, HR-PRO measures are commonly translated into French and administered along with English versions. Scores are pooled together, which implicitly assumes measurement equivalence. When comparisons of French and English versions of HR-PROs are done, they might be best described as parallel examinations of validity and reliability. For example, studies have compared internal consistency statistics [44], [45], convergent validity statistics [45], and factor structures [46]. None of these methods give any indication of whether score metrics are equivalent. The only French study related to the validity of the PHQ-9 that we encountered was conducted in Geneva, Switzerland and was limited to a comparison of PHQ-9 scores and a psychiatrist’s diagnoses of depression [40].

We found only two studies that assessed the measurement equivalence of English and French forms of a HR-PRO measure in a Canadian sample, both of which examined measures of depressive symptoms [47], [48]. One study assessed the measurement equivalence of the Beck Depression Inventory in a sample of adolescents and concluded that there may be important differences between English- and French-speaking adolescents [48]. A second study examined the measurement properties of the Center for Epidemiologic Studies-Depression Scale among caregivers of patients with dementia [47]. That study found minor differences in 4 of 20 items, but concluded that the differences were not large enough to substantively affect scoring. We did not find any studies that have assessed the measurement equivalence of a HR-PRO in medical patients.

Investigation of the cross-linguistic or cross-cultural validity of HR-PROs is important as international collaborations involving multicentre initiatives become increasingly common. Consistent with this, the COSMIN checklist [7] emphasizes the need for cross-cultural comparisons when evaluating HR-PRO measurement tools and specifies that measurement equivalence should be verified when combining data from two different cultural or linguistic groups. Many studies in Canada routinely integrate data from English and French version HR-PROs without first demonstrating that scores are reasonably equivalent across groups. Researchers may assume that cultural or linguistic differences are likely to be insignificant. On the other hand, there are a fairly large number of studies that have assessed the reliability and validity of a HR-PRO in English and French and concluded that the measures can be used interchangeably, but a very small number of studies that have actually assessed measurement equivalence, This would suggest that there is confusion about the degree to which parallel reliability and validity studies establish that HR-PROs in English and French can be used interchangeably and a lack of knowledge of cross-linguistic and cross-cultural measurement techniques. The importance of cross-cultural measurement has become increasingly recognized in medical research in the last 5–10 years, and there are a number of good descriptions of different methods that may be used to examine the cross-cultural equivalence of HR-PROs [49]–[53].

A notable strength of this study includes the use of the MIMIC model in testing for DIF. As outlined in a review evaluating different statistical methods that test for DIF in health settings [54], advantages of the MIMIC model include simultaneous modeling of group differences in the item response and the latent construct, the ability to adjust for the impact of DIF, the ability to include covariates in the model, and that it may be less affected in terms of Type I error than some other methods for assessing DIF that do not use iterative processes to adjust for DIF as the assessment progresses. Disadvantages are that MIMIC models do not assess non-uniform DIF, which is said to occur in cases where DIF may differ at different levels of the latent construct. However this type of DIF is uncommon.

There are limitations that should be considered in interpreting the results of this study. First, it was conducted with a convenience sample of patients with SSc enrolled in the CSRG Registry. Patients with very severe SSc who were too sick to participate, as well as those who may have died earlier in their disease course, are not enrolled in the Registry, which may result in an over-representation of healthier patients. Therefore, the results may be most applicable to relatively stable patients with SSc, and their applicability to other patient groups has not been established. Second, because of the difference in sample size between the English- and French-speaking samples, the core model used to assess DIF relied more on data from English- patients than French-speaking patients. However, since the initial factor analysis yielded the same results in both samples, it does not seem likely that this would have influenced results substantially. Third, there were some differences in sociodemographic and clinical characteristics between the English- and French-speaking samples. However, the sensitivity analysis correcting for differences in demographics between samples yielded virtually the same results as the non-corrected model, which suggests that differences in sample characteristics did not likely influence the results. Finally, a limitation of methods that test for DIF is that results are typically based on statistical significance, as was done in this study. With relatively large samples, such as the sample used in this study, items may reflect statistically significant DIF even with very small magnitude differences between groups [49]. However, assessing the impact of potential DIF on differences in latent factor levels between the two groups, as was done in this study, can help to determine whether DIF would likely influence measurement substantively.

In summary, we found that between French and English versions of the PHQ-9 administered to a Canadian sample with SSc, only 3 items showed measurement invariance, or displayed DIF. These differences were, however, found to be minimal and did not affect the performance of the tool between linguistic groups. The PHQ-9 can be used without adjustment in studies that include Canadian English-speaking and French-speaking SSc patients without undue concern about substantive biases. The analysis of potential DIF in other commonly used HR-PROs should become routine practice prior to using these measures in studies that involve administration in more than one language.
